# Gut Virome: What's the Role in Irritable Bowel Syndrome?

**DOI:** 10.1002/rmv.70080

**Published:** 2025-11-26

**Authors:** Francesco Rettura, Christian Lambiase, Andrea Bottari, Fabio Filippini, Luca Giacomelli, Mauro Pistello, Massimo Bellini

**Affiliations:** ^1^ Gastrointestinal Unit Department of Translational Research and New Technologies in Medicine and Surgery University of Pisa Pisa Italy; ^2^ Gastroenterology Unit Annunziata Hospital AOCS Cosenza Italy; ^3^ Department of Translational Research University of Pisa Pisa Italy; ^4^ Polistudium Srl Milan Italy; ^5^ Department of Translational Research and New Technologies in Medicine and Surgery Retrovirus Center University of Pisa Pisa Italy; ^6^ Virology Unit Pisa University Hospital Pisa Italy

**Keywords:** bacteriophages, dysbiosis, gut microbiota, gut virome, irritable bowel syndrome, phage therapy

## Abstract

The gut virome, an integral but still poorly understood component of the gut microbiota, is emerging as an important player in the pathophysiology of irritable bowel syndrome (IBS). Recent evidence suggests that alterations in virome diversity and phage–bacteria interactions contribute to gut dysbiosis, immune modulation and gut barrier dysfunction in IBS. This review summarises current knowledge on virome alterations in IBS and emphasises the role of bacteriophages in shaping microbial ecology and host responses. Different virome signatures in the different subtypes of IBS highlight the potential of the virome for disease stratification and personalised therapeutic strategies. In addition, we discuss the analytical challenges in virome research and explore novel virome‐targeted interventions, including phage therapy and dietary modulation. A deeper understanding of virome dynamics in the gut could open new avenues for precision medicine approaches to treat IBS.

**Abbreviations**
IBD: inflammatory bowel disease
IBS: irritable bowel syndrome
IBS‐D: diarrhoea‐predominant irritable bowel syndrome
vOTU: viral operational taxonomic unit

## 1 Introduction

The gut is the largest interface between the host and the external environment and plays a crucial role in nutrient absorption, immune development, defence against pathogens and drug metabolism [1]. It harbours a diverse community of commensal, symbiotic and pathogenic microorganisms, collectively referred to as the gut microbiota, whose shared genomes form the gut microbiome [2]. The microbiota consists of approximately 100 trillion cells—more than the number of human cells—and includes bacteria, archaea, fungi, protists and viruses [3, 4].

While the bacterial component of the microbiota has been extensively studied, comparatively little attention has been paid to its viral counterpart, the gut virome, despite its significant impact on gut homoeostasis. Viruses, particularly bacteriophages, outnumber prokaryotes in the gut and actively influence microbial communities through predation, coevolution, horizontal gene transfer and nutrient cycling [5, 6]. These interactions regulate bacterial populations and can have profound effects on the physiology of the host. In diseases such as irritable bowel syndrome (IBS), where gut dysbiosis is a key feature, the virome may play a crucial, as yet undefined role in determining disease progression and symptom variability.

IBS is a chronic disorder of gut–brain interaction, affecting approximately 1.5%–4.1% of the world's population, and is the most common functional gastrointestinal disorder encountered in primary and secondary care [7, 8, 9]. It is characterised by abdominal pain and altered bowel habits without detectable structural or biochemical abnormalities, with a diagnosis based on the Rome IV criteria [10]. The pathophysiology of IBS is multifactorial and includes genetic predisposition, immune disorders, environmental influences, inflammation, visceral hypersensitivity and intestinal dysbiosis [11]. Although dysbiosis is well documented in IBS, it remains unclear whether it is a cause or a consequence of the disease [12, 13]. In addition, microbial disturbances may influence IBS by triggering immune responses, while altered gut motility may in turn promote dysbiosis [14]. Emerging evidence suggests that patients with IBS have reduced gut virome diversity and altered bacteriophage populations, which may affect bacterial composition, gut barrier integrity and immune responses [15]. These findings emphasise the need to further investigate the role of the virome in the pathogenesis of IBS, but this is hampered by several challenges. In contrast to bacteria, viruses lack universal genetic markers, such as the 16S rRNA gene, which makes their identification and classification difficult. A significant proportion of viral sequences identified in metagenomic studies remains unclassified, as viral genomes have limited representation in existing databases [16]. In addition, the distinction between endogenous viruses and transient viruses that are ingested via food or the environment is a major analytical hurdle.

This review summarises the current understanding of the role of the gut virome in the pathophysiology of IBS, highlights the analytical challenges, and explores potential diagnostic and therapeutic applications. Understanding the interactions of the gut virome with the bacterial microbiome and the host immune system could provide new insights into the aetiology of IBS and open up new therapeutic opportunities, such as targeted bacteriophage therapies and microbiome‐based interventions.

While this review focuses on the virome's role in IBD and IBS, it is important to emphasise that linking the gut virome to disease states remains particularly challenging. The technical and conceptual hurdles described above—from taxonomic uncertainty and sparse data to low abundance and annotation limitations—all contribute to the difficulty of establishing robust virome‐disease associations. Consequently, even when apparent virome alterations are detected in disease contexts, their interpretation and causal inference remain speculative. We are only beginning to catalogue viral diversity, and dedicated frameworks for virome‐wide association studies are still emerging [17].

## 2 Gut Virome

The gut virome, composed mainly of bacteriophages, plays a key role in shaping the bacterial microbiome by influencing microbial composition, genetic exchange and metabolic function [18]. Despite its importance, a large proportion of enteric viruses are still unclassified due to the lack of universal viral markers and the limited representation of viral genomes in reference databases [19, 20]. Unlike bacteria, which can be phylogenetically classified using the highly conserved 16S rRNA gene, viruses lack a universal genetic marker across taxa. This limits the ability to assign taxonomic identities to viral sequences, especially for novel or divergent species, and complicates phylogenetic reconstruction and ecological comparisons [21].

Key repositories, such as the Gut Virome Database (GVD), Metagenomic Gut Virus (MGV) catalogue, and Gut Phage Database (GPD), have greatly expanded known viral diversity and improved profiling of gut viromes. However, these databases remain incomplete. A significant portion of viral sequences still lacks taxonomic assignment, host prediction remains limited, and RNA viruses are notably underrepresented [21]. This complicates the annotation of the virome and leads to a significant proportion of unidentified reads in metagenomic studies [16]. Geographic bias is another issue, with most database entries originating from Western populations, despite increasing IBD and IBS incidence in other regions [22]. In addition to database limitations, taxonomic classification of viruses remains a major analytical challenge. The coexistence of different systems—such as the ICTV's hierarchical taxonomy and the Baltimore classification based on replication strategy—often leads to inconsistent or conflicting assignments [21]. The adoption of metagenomics has led to the identification of thousands of novel viruses, many of which lack proper descriptions, standardized nomenclature, or clear phylogenetic placement [17]. Some higher‐level taxa, including several families, are known to be polyphyletic, undermining their taxonomic stability [17]. These issues complicate cross‐study comparisons and hinder the development of robust, universally applicable virome markers. Ongoing efforts by ICTV to incorporate genomic data into viral taxonomy may help resolve some of these discrepancies.

Bioinformatic pipelines present additional obstacles. Most established tools were originally developed for bacterial metagenomics and perform poorly on viral datasets due to fundamental differences in genome structure, gene content, and evolutionary dynamics [23]. Standard workflows often rely on homology‐based methods, which fail when viral proteins lack characterised homologues. Moreover, statistical models such as those used in diversity analysis or network inference struggle with the high sparsity of viral presence‐absence matrices across samples [24].

Recent developments offer some promise. Approaches such as GOPhage incorporate deep learning and genomic context to annotate viral proteins that lack homology, achieving improved performance even on poorly characterised phages [25]. These tools outperform traditional pipelines in sensitivity and annotation depth, especially for low‐abundance or taxonomically novel viruses. Continued adaptation of virus‐specific bioinformatics solutions will be essential for robust virome analysis.

Another major obstacle is the typically low abundance of viral nucleic acids in gut samples. Even in virus‐enriched preparations, host and bacterial DNA can dominate, reducing the sensitivity of virus detection [21, 23]. This imbalance affects both sequencing depth and the accuracy of bioinformatic classification, particularly for rare or low‐copy‐number viruses. Bulk metagenomic approaches are especially prone to this issue, while virus‐like particle enrichment can help but may not fully eliminate background contamination [22]. As a result, both false negatives and underestimations of viral diversity are common in virome studies.

However, recent advances in next‐generation sequencing and bioinformatics tools have improved virome analysis and increased the ability to profile viral communities. Tools, such as VIROMEScan, Metavir, PHACCS and the VIROME pipeline, integrate multiple sequence databases to extend the analysis beyond the boundaries of individual datasets and facilitate the assembly and classification of viral genomes [26, 27, 28, 29, 30, 31, 32, 33, 34].

The gut virome is estimated to consist of approximately 10^9^ viral particles per gram of faeces [35] and is composed primarily of prokaryotic viruses (97.7%), with smaller proportions of eukaryotic (2.1%) and archaeal viruses (0.1%) [6, 36]. Most of these viruses belong to the order Caudovirales, which includes families, such as the Myoviridae, Podoviridae and Siphoviridae, but other families, such as the Microviridae and Inoviridae, also contribute to the diversity of the virome [36, 37, 38, 39]. Phages regulate bacterial populations through predation, horizontal gene transfer and lysogeny and thus influence intestinal homoeostasis [4, 40].

Both bacteriophages and eukaryotic viruses have lytic and lysogenic life cycles, with environmental factors influencing the transition between these states [41, 42, 43]. These dynamics are critical for maintaining microbial balance in the gut and may have implications for gastrointestinal disorders, such as IBS [44].

## 3 Virome Interactions With the Bacteriome

Recent studies show the intricate relationship between the virome and the bacteriome, with phages playing a crucial role in shaping bacterial populations [45]. Large‐scale metagenomic analyses show significant correlations between viral and bacterial diversity, suggesting that the virome is actively involved in regulating the microbiome and does not merely exist as passive genetic material [46, 47, 48].

The virome exhibits higher inter‐individual variability than the bacteriome, suggesting a personalised composition of the virome [45, 49, 50]. Certain genera, such as *Megamonas*, *Escherichia*, *Prevotella* and *Lactobacillus*, are strongly associated with their respective phages, indicating a specialised phage‐host relationship. In contrast, generalist phages that infect multiple bacterial genera, such as *Clostridium*, *Ruminococcus* and *Tyzzerella*, contribute to a broader modulation of the microbiome [45].

While phages typically kill their bacterial hosts, leading to the expected negative correlations, several studies reveal patterns of co‐occurrence that emphasise the limited host range and symbiotic nature of phage‐bacteria relationships [51, 52, 53, 54]. Host distribution appears to be a critical factor for phage dissemination in the gut ecosystem [45].

Bacteria have evolved antiviral defence mechanisms, such as CRISPR‐Cas systems, restriction‐modification systems, and toxin–antitoxin modules that can prevent excessive lysis of certain bacterial populations and thus influence virome composition and phage–host interactions [55]. Genes associated with integrase and spore germination, particularly in *Bacillota* species, also influence virome diversity [56]. Protection against phage infection may influence bacteriome structure by preventing excessive lysis of specific bacterial populations [45, 52].

In addition to bacterial interactions, host factors, such as age, medication and disease status, also correlate with the composition of the virome [45]. However, the variability of the virome associated with these factors is less than one compared to 5%–10% for the bacteriome, highlighting the complexity and adaptability of viral communities in the gut [57, 58]. Such diversity may arise from rapid phage evolution variability in bacterial strain and their defence systems [49, 59, 60]. Understanding the mechanisms and the factors driving variability has important clinical implications for interventions, such as faecal microbiota transplantation and phage therapy, which aim to restore gut microbial balance [61]. However, further efforts are needed to establish comprehensive phage catalogues. Some newly identified RNA phage clades target Firmicutes and Actinobacteria, in contrast to previously described crAss‐like phages that predominantly infect Bacteroidetes [62, 63].

### 3.1 Gut Virome and Its Impact on Host Physiology

The gut virome goes beyond microbial communities and influences the physiology of the host through interactions with the immune system and modulation of the intestinal barrier [64, 65, 66, 67, 68]. The intestinal mucosa, which consists of epithelial cells, immune cells and the microbiota, plays a crucial role in maintaining immune homoeostasis [64, 69]. Disruptions to this balance contribute to various diseases, including inflammatory bowel disease (IBD) [70], IBS [71, 72], and metabolic or autoimmune diseases [73, 74, 75, 76, 77, 78]. Genetic predisposition, environmental factors (e.g., stress, diet, toxins, drugs) and microbial imbalances, including viruses, can compromise the mucosal barrier and further promote disease [64, 65, 66, 67, 68, 79].

Recent evidence suggests that virome alterations in IBD [80, 81, 82] and colorectal cancer [83] may contribute to disease‐specific virome alterations. In IBS, studies suggest reduced virome *α*‐diversity and different *β*‐diversity patterns compared to healthy controls [3, 84]. These shifts could affect intestinal barrier integrity, immune responses and microbial stability, all of which are implicated in the pathogenesis of IBS [11, 85].

### 3.2 Bacteriophages

As important regulators of bacterial populations, bacteriophages can have profound effects on gut health [86]. In addition to their role in bacterial predation, phages also contribute to intestinal immune homoeostasis by improving the integrity of the mucosal barrier, thereby preventing bacterial translocation and modulating inflammatory responses [87, 88]. For example, phages interact with the intestinal mucus, prevent bacterial overgrowth and maintain the integrity of the epithelium. Conversely, phage‐encoded proteins play important roles in bacterial virulence, allowing adhesion, invasion of the gut barrier [89, 90], production or release of exotoxins [91], and counteracting phagocytic activity that otherwise would ensure immune clearance [92].

An imbalance in the phage community can disrupt immune homoeostasis and contribute to chronic diseases, such as IBS, IBD and metabolic disorders [70, 73, 84, 93, 94]. The mechanisms underlying these effects are still poorly understood, highlighting the need for further research into the immunological consequences of phage‐host interactions [78, 95].

At the intestinal mucosa, phages can directly penetrate the epithelial barrier through transcytosis, accessing circulation [96, 97, 98] and interacting with antigen‐presenting cells and host immune receptors, such as Toll‐like receptors, NOD‐like receptors and RIG‐I‐like receptors, thus triggering innate immune responses [99, 100, 101, 102, 103]. These interactions are tightly regulated to prevent inappropriate immune activation against commensal phages [104].

While phages have been shown to elicit humoural anti‐phage immune responses [105], their broader immunomodulatory effects are still poorly understood. Some studies suggest that phages may induce long‐term immune tolerance by interacting with regulatory T cells and promoting intestinal immune stability [104, 106]. Conversely, aberrant phage transcytosis across the epithelial barrier may increase inflammation and thus contribute to disease pathology [98].

### 3.3 Eukaryotic Viruses

Eukaryotic viruses also play an important role in intestinal homoeostasis and immune regulation. In mouse models, it has been shown that depletion of intestinal viruses can lead to intestinal inflammation, while the presence of specific viral ligands attenuates disease progression [107]. These viruses interact with gut bacteria in complex ways, although the mechanisms governing these relationships are poorly understood [108, 109].

**FIGURE 1:**
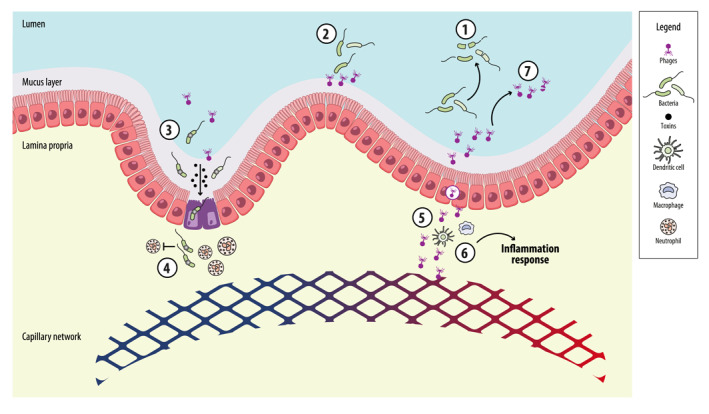
Potential interaction between gut microbiota (i.e., bacteriophages, eukaryotic viruses, and bacteria) and human cells (e.g., enterocytes and immune cells). Reciprocal interactions between these components may have important consequences for our gut homoeostasis. Phages regulate bacterial populations through 1 predation, horizontal gene transfer and lysogeny (1) and provide a barrier effect against bacterial colonisation of the gut barrier (2). Phage‐encoded proteins play important roles in bacterial virulence, allowing for adhesion, invasion of the gut barrier, toxin production (3), and inhibiting neutrophil phagocytosis (4). Phages can directly penetrate the epithelial barrier through transcytosis (5), accessing circulation and interacting with antigen‐presenting cells and host immune receptors (6) (e.g., Toll‐like receptors, NOD‐like receptors, and RIG‐I‐like receptors), thus triggering innate immune responses. Bacteria have evolved antiviral defence mechanisms (7) (e.g., CRISPR‐Cas systems, restriction‐modification systems, and toxin‐antitoxin modules) to prevent excessive lysis. The role of eukaryotic viruses in this complex network is still under debate. However, they are rich in functional pathways related to signal transduction, metabolic enzyme activity and modulation of the host immune system.

Eukaryotic viruses are rich in functional pathways related to signal transduction, metabolic enzyme activity and modulation of the host immune system, suggesting that they have a broader impact on the dynamics of the gut microbiome [110]. Similar to commensal bacteria, these viruses contribute to gut stability and immune regulation, emphasising the need for virus‐focused research in IBS and other gastrointestinal disorders (Figure 1) [111].

Beyond overt gastroenteritis, eukaryotic enteric viruses can modulate gut physiology through direct epithelial tropism and tonic type I/III interferon signalling, which in turn influences barrier integrity, antimicrobial peptide production, and downstream adaptive responses; these effects are strongly context dependent and can be either protective or pro‐inflammatory [112].

SARS‐CoV‐2 offers a recent clinical example linking non‐phage viral infection to longer‐term gut outcomes: hospitalised patients with COVID‐19 displayed substantial acute GI involvement with persistence of symptoms at follow‐up [113], and a prospective study reported a higher 12‐month incidence of new‐onset IBS compared with controls [114].

Mechanistically, sustained mucosal immune activation, epithelial ACE2‐related metabolic perturbations (e.g., tryptophan handling), and post‐infectious dysbiosis have been proposed to connect eukaryotic viral infections to chronic gastrointestinal sequelae, including IBS; recent syntheses of post‐COVID digestive manifestations support these pathways while noting variability across cohorts [115].

## 4 Current and Potential Role of the Gut Virome in IBS

**FIGURE 2:**
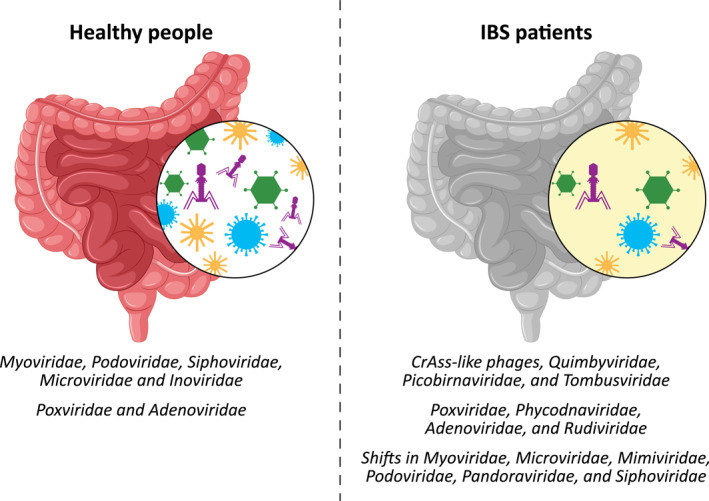
Schematic illustration of the putative differences in viral composition (phages and eukaryotic viruses) in irritable bowel syndrome (IBS) in comparison to healthy people. The gut virome in healthy people modulates bacterial populations, host immunoinflammatory responses, and the integrity of the mucosal barrier by promoting intestinal homoeostasis. Conversely, the putative role of gut virome alterations in IBS patients is to compromise the mucosal barrier, trigger immuno‐inflammatory responses, induce gut dysbiosis through phage‐mediated virulence increase, and eukaryotic virus‐mediated epithelial metabolic perturbations.

There is increasing evidence that the intestinal virome plays an important role in the pathophysiology of IBS, with significant differences in the viral composition of IBS and healthy controls (Table 1; Figure 2). Recent studies suggest that IBS is characterised by reduced viral diversity, shifts in bacteriophage populations and altered virus‐bacteria interactions that may contribute to gut dysbiosis and disease progression.

**TABLE 1:** Overview of gut virome alterations in IBS patients compared to healthy controls.

Reference	Comparison	Increased	Decreased	Conclusion	Method
Ansari et al. [26]	IBS (*n* = 25) versus HC (*n* = 17)	*Pandoravirus salinus*	*Pandoravirusinopinatum,* Poxviridae*,* Phycodnaviridae*,* Adenoviridae*,* Rudiviridae	Viral taxa may serve as biomarkers or therapeutic targets in IBS	Metagenomic analysis
Coughlan et al. [84]	IBS (*n* = 55) versus HC (*n* = 51)	1 VC in Mimiviridae*,* Podoviridae*,* Siphoviridae	1 VC in Mimiviridae*,* Podoviridae, and 2 VCs in Siphoviridae	Virome in IBS differs from controls, indicating potential for therapeutic development	Metagenomic sequencing
Mihindukulasuriya et al. [5]	IBS‐D (*n* = 17) versus HC (*n* = 16)	1 species each in Microviridae*,* Myoviridae*,* Podoviridae	/	Gut virome remains stable but interacts with bacterial composition and diet	Virus‐like particle metagenomic sequencing
	IBS‐C (*n* = 17) versus HC (*n* = 16)	2 species in Microviridae, 1 species in Siphoviridae	/	Virome stability influences gut function	Metagenomic sequencing
	IBS‐D versus IBS‐C (*n* = 17 each)	1 species each in Microviridae*,* Myoviridae*,* Siphoviridae, and 2 in Podoviridae	3 species in Microviridae, 1 in Myoviridae	Virome variation correlates with IBS subtype differences	Metagenomic sequencing
Zhang et al. [15]	IBS (*n* = 277) versus HC (*n* = 84)	111 vOTUs (*crAss‐like,* Siphoviridae*,* Myoviridae*, Quimbyviridae*)	16 vOTUs (multiple families)	Distinct virome profiles and vOTU abundance differentiate IBS from controls	Fecalmetagenomics
Xie et al. [116]	IBS‐D (*n* = 50) versus HC (*n* = 30)	Siphoviridae*,* Podoviridae*,* Microviridae*,* Picobirnaviridae*,* Tombusviridae	/	Unique DNA and RNA virome signatures in IBS‐D, linked to metabolite and bacterial shifts	Multiomic analysis
Li et al. [110]	IBS (*n* = 37) versus HC (*n* = 18)	Siphoviridae*,* Myoviridae*,* Podoviridae	/	Viral shifts correlate with bacterial/metabolite changes, highlighting phage–bacteria interactions	Metagenomics and metabolomics

Abbreviations: HC, healthy controls; IBS, irritable bowel syndrome; IBS‐C, constipation‐predominant irritable bowel syndrome; IBS‐D, diarrhoea‐predominant irritable bowel syndrome; VC, viral cluster; vOTU, viral operational taxonomic unit.

A recurrent finding is a reduction in overall viral diversity and richness in IBS patients compared to healthy controls. In one of the first faecal virome analyses, Ansari et al. identified significantly reduced viral diversity in IBS, with specific differences in Pandoravirus species and an enrichment of virus families such as Poxviridae and Adenoviridae in controls, suggesting possible protective roles [3]. Coughlan et al. confirmed a similar trend of reduced phage alpha diversity, also highlighting the highly individualised nature of the virome in IBS [84].

In terms of virome composition and taxonomic shifts, several studies have described distinct patterns. While common viral families such as Myoviridae, Podoviridae and Siphoviridae were found across both IBS and control groups [3, 84], differences emerged at finer taxonomic levels. Coughlan et al. reported increased abundance of unclassified viruses from Mimiviridae and other families in IBS patients, while observing that lytic phages remained predominant, in contrast to shifts towards temperate phages often seen in IBD [84]. Similarly, Li et al. observed phage population shifts involving Siphoviridae, Myoviridae and Podoviridae, with functional viral genes linked to host transcription and metabolism [110]. Xie et al. further reported differential profiles in both DNA and RNA viruses among IBS‐D patients, particularly affecting families, such as Microviridae and Picobirnaviridae [116].

Some studies have provided insight into IBS subtype‐specific virome signatures. For instance, phages infecting *Lactobacillus* were more prevalent in constipation‐predominant IBS (IBS‐C), whereas unclassified phages from Microviridae and Podoviridae were enriched in IBS‐D [5]. These subtype differences may reflect underlying microbial and metabolic distinctions between IBS phenotypes.

Emerging data also point to associations between the virome, bacterial dysbiosis, and host metabolism. Li et al. reported that the depletion of beneficial genera such as *Lactobacillus* and *Lactococcus* correlated with a reduction in their corresponding phages, suggesting a lysogenic relationship that may support gut homoeostasis [110]. Network analyses by Xie et al. revealed fewer virus–bacteria–metabolism interactions in IBS‐D compared to controls, especially involving short‐chain fatty acid producers [116].

Finally, a large‐scale analysis involving 277 IBS patients and 84 controls identified over 100 virome operational taxonomic units enriched in IBS, including crAss‐like phages and *Quimbyviridae*. These IBS‐enriched virome operational taxonomic units correlated with disease‐associated bacteria such as *Klebsiella pneumoniae* and *Ruminococcus gnavus*, underscoring the complex interplay between viruses and bacteria in IBS pathophysiology [15].

## 5 Expert Opinion

The connection between intestinal bacteria and IBS is well known. Numerous studies have shown changes in the microbiome in IBS patients [117]. A consistent finding is an increased ratio of Firmicutes‐to‐Bacteroidetes, along with increased *Bacteroides* and reduced *Bifidobacterium* [118, 119, 120, 121]. These shifts in microbial composition reflect broader patterns of dysbiosis associated with IBS symptoms. Certain bacterial species, such as enterotoxigenic *Bacteroides fragilis*, exacerbate symptoms by degrading glycoproteins in the gut and disrupting motility, leading to diarrhoea and abdominal pain [122]. Conversely, beneficial microbes such as *Bifidobacterium infantis* can alleviate the symptoms of IBS by regulating inflammatory responses and promoting intestinal homoeostasis [123, 124].

Despite extensive research on bacterial dysbiosis in IBS, the role of the virome remains largely unexplored [125]. This gap in knowledge represents a major clinical limitation. There are currently no pharmacological treatments that target or modulate the gut virome, although viral communities may play a crucial role in the pathophysiology of IBS. The possibility that probiotics may indirectly affect the virome is a compelling but as yet unproven hypothesis. It raises an important question: Is there a ‘pathological virome’ associated with bacterial dysbiosis in IBS, and could it be used as a therapeutic target?

The intestinal phageome, the predominant viral component of the microbiota, is particularly important in this context. Early attempts to characterise the human virome, such as the study by Manrique et al., showed a multilevel distribution of bacteriophages. They identified a core phageome (e.g., *crAssphage*, present in more than half of individuals), a community group (shared by 20%–50% of individuals) and a single group of phages present in only a small subset of the population [126]. Interestingly, the majority of the virome consists of unique phages, making it difficult to define a universal virome signature in IBS. However, certain phages, such as *crAssphage,* may be fundamental to the gut microbial ecology regardless of health status [127].

One particularly intriguing possibility is that phages influence the efficacy of probiotics, which could explain the variability observed in the treatment of IBS. Although *Lactobacillus* and *Bifidobacterium* probiotics are commonly used to treat IBS, their effectiveness varies from person to person. This variability could be due to the different composition of the virome, which either favours or inhibits colonisation with probiotics. For example, lysogenic phages that target beneficial bacteria may reduce the effectiveness of probiotics, while others may facilitate colonisation by eliminating competing bacterial strains.

This hypothesis is supported by findings showing that *Levilactobacillus brevis* KB290 administration improved IBS symptoms and increased *Bifidobacterium* levels while reducing *Clostridium*. However, in some patients, free lysogenic phages (LBR‐48) targeting *L. brevis* reduced the efficacy of the probiotic, suggesting that phage predation may be a key factor in the success or failure of probiotics [5, 128]. Understanding how the virome modulates bacterial populations could ultimately lead to more personalised and effective microbiome‐targeted therapies for IBS.

In addition to probiotics, dietary interventions may be another way to indirectly modulate the gut virome. Phage populations fluctuate in response to changes in diet, and some of these shifts correlate with improved gut health. In contrast, virome perturbations are commonly observed in diseases such as obesity and malnutrition [129, 130, 131], raising the possibility that nutritional strategies aimed at enriching beneficial bacteriophages could influence gut health. Such interventions could increase the efficacy of probiotics or restore microbial balance in patients with irritable bowel syndrome [132, 133, 134].

However, viral research still faces major challenges in IBS. The limited availability of viral reference databases and the lack of sensitive detection methods hinder our ability to fully characterise the virome's contribution to gut dysbiosis [16]. Furthermore, the highly personalised nature of the virome makes it difficult to generate universal viral signatures for IBS. Overcoming these challenges will require improvements in sequencing technology, enhanced bioinformatics pipelines and expanded virome studies to uncover the functional role of gut viruses in IBS.

A promising approach to direct virome modulation is phage therapy. Although still in its infancy, phage administration has been shown to normalize levels of inflammatory cytokines, reduce TNF‐α and IL‐6, while triggering anti‐inflammatory responses via IL‐1 receptor antagonists and upregulation of SOCS3 [135]. These results suggest that targeted phage therapy could help to regulate intestinal inflammation, providing an innovative and precise approach for the treatment of IBS.

## 6 Conclusion

Although the gut virome is less studied than the bacterial components of the microbiota, there is evidence that it plays a critical role in gut health and disease, including IBS. Differences in phage diversity, abundance and interactions with the bacterial microbiome may contribute to the pathophysiology of IBS, but these viral changes are not yet well understood. The role that the virome plays in shaping bacterial communities, immune responses, and gut homoeostasis underscores its potential as a novel diagnostic and therapeutic target in IBS.

Given the variable efficacy of probiotics, it is plausible that the virome plays a crucial role in treatment success. Future research should aim to characterise virome changes in different subtypes of IBS, identify functional interactions between virome and bacteriome, and investigate how dietary and therapeutic interventions influence virome dynamics. Expanding virome research and integrating it into broader microbiota studies will be critical for the development of more effective, personalised IBS treatments.

Further development of sequencing technologies and bioinformatics tools to improve the detection and classification of viral genomes, studies investigating phage‐probiotic interactions and bridging the gap between virome and microbiome research can pave the way for innovative strategies to regulate gut inflammation and dysbiosis and ultimately improve patient outcomes.

## Author Contributions

Study conception and design: F.R., C.L., M.B. Collection and interpretation of data: A.B., F.F. Manuscript draughting: F.R., C.L. Manuscript editing: L.G. Approval to submit: M.P., M.B.

## Funding

This project was supported by the Italian Ministry of University and Research (MUR), awarded to M.P. under the PRIN2022 program (Grant 2022FRE3RH), “*Dissecting the host cellular response to develop novel host‐targeted approaches against RNA viruses*” (CUP I53D23000480006), within the National Recovery and Resilience Plan (PNRR), Mission 4 “Education and Research,” Component 2, Investment 1.1.

## Ethics Statement

The authors have nothing to report.

## Consent

The authors have nothing to report.

## Conflicts of Interest

The authors declare that the research was conducted in the absence of any commercial or financial relationships that could be construed as a potential conflict of interest.

## Data Availability

The authors have nothing to report.
